# Terminal group engineering of A–DA′D–A non-fullerene acceptors as electron transport materials for efficient inverted perovskite solar cells

**DOI:** 10.1039/d6ra02579j

**Published:** 2026-05-19

**Authors:** Shengdong Zhao, Yufei Gong, Fei Pan, Junjiang Guo, Chengcheng Xie, Chenxing Lu, Xiaojun Li, Jingchao Xu, Menglan Lv

**Affiliations:** a Engineering Research Center for Energy Conversion and Storage Technology of Guizhou, School of Chemistry and Chemical Engineering, Guizhou University Guiyang 550025 PR China mllv@gzu.edu.cn; b Beijing National Laboratory for Molecular Sciences, CAS Key Laboratory of Organic Solids, Institute of Chemistry, Chinese Academy of Sciences Beijing 100190 PR China lixiaojun@iccas.ac.cn; c School of Chemical Engineering, Guizhou Institute of Technology Guiyang 550025 PR China fpan@git.edu.cn; d Dongfang Electric (Hangzhou) Innovation Institute Co., Ltd Hangzhou PR China xc2009xust@163.com

## Abstract

Conventional fullerene-based electron transport materials (ETMs) suffer from limited tunability and suboptimal interfacial contact, hindering further efficiency improvements in inverted perovskite solar cells (PSCs). In this study, two non-fullerene small molecules with chlorinated thiophene terminal groups Cl24-TCl and difluorinated phenyl terminal groups Cl24-F were employed as ETMs in inverted PSCs to investigate the key factors by which different terminal groups influence device performance. Although the Cl24-TCl ETM exhibits a more polarized electrostatic potential distribution and a larger dipole moment, the Cl24-F ETM shows more favorable energy level alignment and interfacial contact, which effectively reduce interfacial charge recombination losses. In addition, Cl24-F displays more compact π–π stacking, resulting in higher electron mobility and conductivity, thereby promoting more efficient interfacial charge transport. Consequently, inverted PSCs based on Cl24-F achieve a champion efficiency of 24.18%, which is significantly higher than that of devices based on Cl24-TCl (13.02%). This work uncovers the key factors governing device performance and guides the design of high-performance non-fullerene ETMs.

## Abstract 2

Terminal group engineering in A–DA′D–A acceptors enables efficient electron transport, with Cl24-F achieving 24.18% *via* optimized energy alignment and charge dynamics.

## Graphical abstract

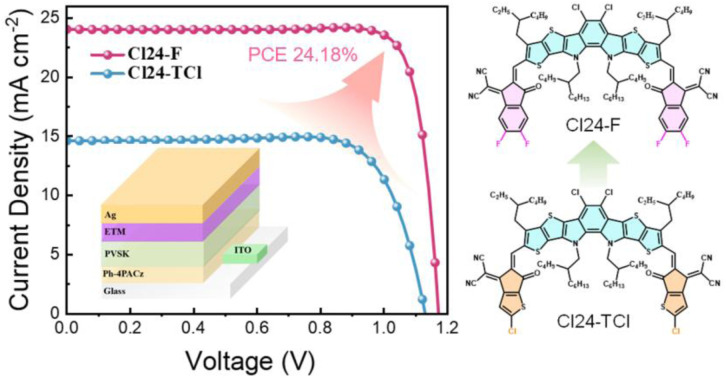

## 1 Introduction

Inverted (p-i-n) perovskite solar cells (PSCs) have attracted considerable research interest owing to their low-temperature processability, negligible hysteresis, and compatibility with tandem device architectures.^1–4^ Over the past decade, the power conversion efficiencies (PCEs) of inverted PSCs have surged dramatically, recently exceeding 27% in single-junction devices.^5^ Among the critical components governing device performance, the electron transport materials (ETMs) play an indispensable role in extracting and transporting photogenerated electrons from the perovskite absorber to the cathode.^6,7^ Fullerene derivatives have long served as the benchmark ETMs for inverted PSCs due to their suitable energy levels and high electron mobility.^8,9^ However, fullerenes suffer from poor solubility, pronounced thermal aggregation, restricted energy level tunability, and high synthesis costs.^10,11^ Such limitations have driven the search for non-fullerene ETMs that offer improved stability, tunable electronic properties, and versatile molecular design.^12,13^

Among the emerging non-fullerene ETMs, Y-series A–DA′D–A molecules, originally developed for organic photovoltaics,^14^ show great potential as ETMs in inverted PSCs.^15–17^ With broad absorption, high electron mobility, and minimized voltage loss, these molecules promote efficient charge extraction. Meanwhile, their multifunctional groups passivate perovskite surface defects, reducing interfacial recombination.^16,18^ Recent studies have demonstrated that Y-series non-fullerene acceptors can achieve PCEs exceeding 25% when employed as ETMs, rivaling the performance of fullerene-based devices.^16,19^ The rational engineering of terminal electron-withdrawing groups has emerged as a powerful strategy to fine-tune the energy levels, molecular packing, and interfacial interactions of A–DA′D–A-type ETMs. For instance, Liu *et al.* systematically modulated the LUMO levels of ITIC-series small molecules (IT-4F, IT-4H, IT-4M) by introducing substituents with different electronegativities on the terminal phenyl groups, and demonstrated that the up-shifted LUMO of IT-4M yielded a larger built-in potential and consequently a superior open-circuit voltage (*V*_OC_) and PCE in inverted PSCs.^20^ In parallel, Hu *et al.* developed a spiro-based non-fullerene ETM SPS-4F end-capped with fluorinated 1,1-dicyanomethylene-3-indanone units, which exhibited a high electron mobility and effective trap passivation, yielding a PCE exceeding 20% with significantly improved ambient stability.^21^ Feng *et al.* recently reported a cyano-functionalized bithiophene imide-based polymer ETM (PCNI2-BTI) which achieved a certified efficiency of 25.4% in inverted PSCs. The cyano terminal groups not only deepened the LUMO level for efficient electron extraction but also provided strong Lewis base passivation of undercoordinated Pb^2+^ defects at the perovskite surface.^22^ Collectively, these advances underscore that terminal group chemistry dictates not only the molecular electrostatic properties but also the thin-film microstructure and perovskite/ETM interfacial charge dynamics, which are pivotal for maximizing device performance. Despite this encouraging progress, a direct comparative study elucidating how different terminal acceptor units on the same A–DA′D–A core govern the interplay between molecular polarity, film morphology, and interfacial recombination remains lacking.

In this work, we employed two A–DA′D–A non-fullerene acceptors, Cl24-TCl and Cl24-F, as ETMs in perovskite solar cells. The molecules bore different terminal electron-withdrawing groups and yielded champion PCEs of 13.02% and 24.18%, respectively. The effects of terminal group variations on molecular dipole moments and electrostatic potentials were characterized. Integrating these findings with analyses of film morphology, energy level alignment, charge transport, and interfacial carrier dynamics establishes a clear relationship between terminal group structure and device performance. This work identifies the key factors governing device efficiency and provides valuable guidance for the rational design of high-performance ETMs.

## 2 Results and discussion

### 2.1 Molecular design and electrostatic properties

**Fig. 1:**
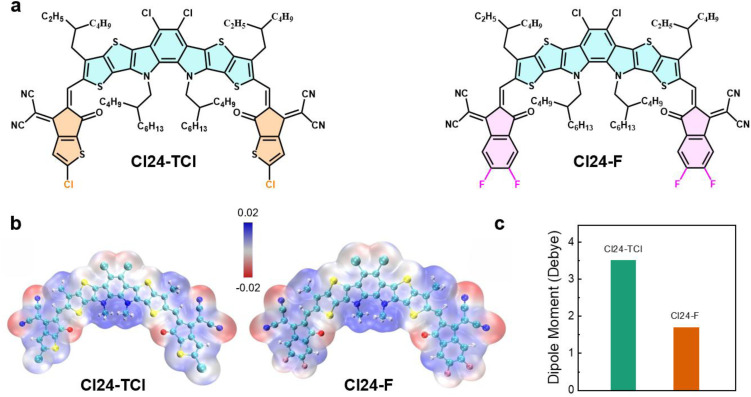
(a) Chemical structures, (b) electrostatic potential maps, (c) dipole moment of the Cl24-TCl and Cl24-F.

The molecular structures of Cl24-TCl and Cl24-F are illustrated in Fig. 1a. Both molecules adopt an A–DA′D–A molecular framework, featuring a central electron-deficient core flanked by electron-donating units and terminated with strong electron-withdrawing groups. The key structural difference lies in the terminal acceptor units. Cl24-TCl employs chlorine-substituted thiophene, while Cl24-F incorporates difluorinated benzene.

To investigate the effect of the terminal groups on the electronic properties, density functional theory (DFT) calculations were performed (Fig. 1b). The electrostatic potential (ESP) reveals distinct charge distribution patterns for the two molecules. Cl24-TCl exhibits more pronounced charge separation, with electron-deficient regions concentrated around the terminal chlorine atoms and electron-rich regions localized on the central core. In contrast, Cl24-F displays a more balanced charge distribution, with the fluorine atoms contributing to a more diffuse electron-withdrawing effect. Quantitative ESP analysis (SI Table 1) provides further insights into the molecular charge distribution characteristics. Cl24-TCl exhibits an average ESP of 11.82 kJ mol^−1^, variance of 134.19 kJ^2^ mol^−2^, charge parameter of 0.25, and polarity index of 11.29 kJ mol^−1^, whereas Cl24-F exhibits corresponding values of 4.55 kJ mol^−1^, 131.43 kJ^2^ mol^−2^, 0.24, and 10.46 kJ mol^−1^. These parameters indicate that Cl24-TCl has stronger local charge separation while Cl24-F exhibits a more balanced charge distribution. The dipole moments were calculated to be 3.50 D for Cl24-TCl and 1.70 D for Cl24-F (Fig. 1c). The higher dipole moment of Cl24-TCl arise from the electron-withdrawing effect and asymmetric charge distribution of its chlorine-substituted thiophene unit, which may induce stronger intermolecular interactions and adversely affect molecular packing and film morphology.^23^

### 2.2 Photovoltaic performance

**Fig. 2:**
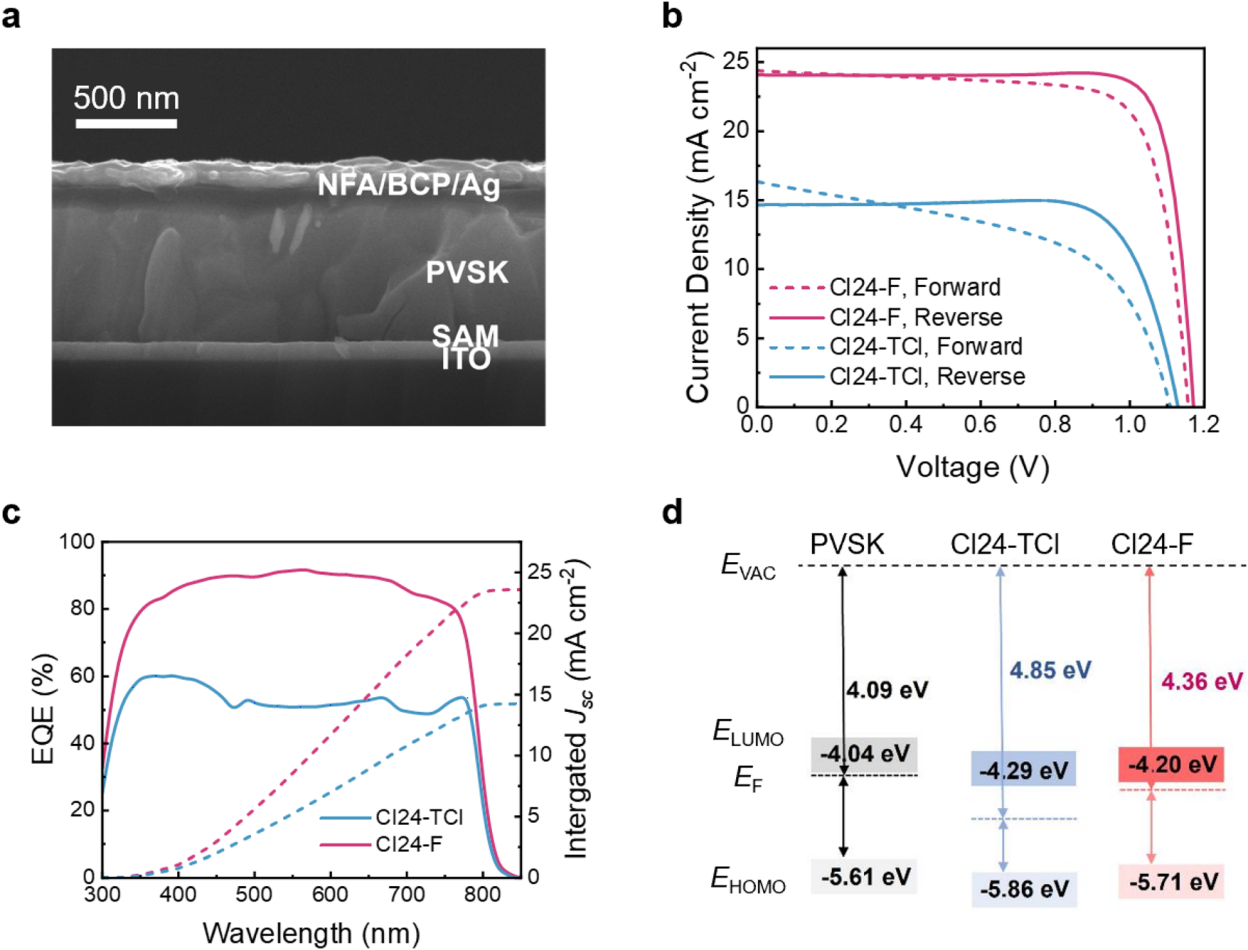
(a) Cross-sectional images of PSCs, (b) optimized *J*–*V* curves and (c) EQE spectra with the integrated *J*_SC_ values of the PSCs, (d) energy level of PVSK, Cl24-TCl, and Cl24-F.

To evaluate the impact of terminal group engineering on device performance, inverted PSCs with the architecture ITO/SAM/perovskite/ETM/BCP/Ag were fabricated with Cl24-TCl or Cl24-F as the ETM (Fig. 2a). The Cl24-F-based device achieves an outstanding reverse-scan PCE of 24.18%, with a *V*_OC_ of 1.171 V, a short-circuit current density (*J*_SC_) of 24.56 mA cm^−2^, and a fill factor (FF) of 84.09% (Fig. 2b and Table 1). The forward scan shows a PCE of 21.96%, with a *V*_OC_ of 1.157 V, a *J*_SC_ of 24.87 mA cm^−2^, and an FF of 76.31%, indicating a small hysteresis index (HI) of 9.18%, which is typical for well-optimized inverted PSCs. In contrast, the Cl24-TCl-based device exhibits significantly inferior performance, with a reverse-scan PCE of only 13.02%, a *V*_OC_ of 1.136 V, a *J*_SC_ of 14.84 mA cm^−2^, and an FF of 77.26%, and a forward-scan PCE of 6.18%, a *V*_OC_ of 1.126 V, a *J*_SC_ of 11.17 mA cm^−2^, and an FF of 49.14%, showing pronounced hysteresis, indicative of poor interfacial charge transfer and severe trap-mediated recombination.^24^ To benchmark the non-fullerene ETMs against the conventional fullerene counterpart, inverted PSCs employing PCBM as the ETM were also fabricated under identical conditions. The champion PCBM-based device yields a PCE of 20.92%, with a *V*_OC_ of 1.161 V, a *J*_SC_ of 23.54 mA cm^−2^, and an FF of 76.55% (SI Fig. 1 and Table 2), which is substantially inferior to that of the Cl24-F-based device.

**Table 1:** Champion photovoltaic performance parameters of PSC devices based on different ETMs

ETMs	*V*_OC_ (V)	*J*_SC_ (mA cm^−2^)	FF (%)	PCE (%)	*J*_cal_ a (mA cm^−2^)	HI^b^ (%)
Cl24-TCl (forward)	1.126	11.17	49.14	6.18	14.25	52.53
Cl24-TCl (reverse)	1.136	14.84	77.26	13.02		
Cl24-F (forward)	1.157	24.87	76.31	21.96	23.58	9.18
Cl24-F (reverse)	1.171	24.56	84.09	24.18		

a The values were determined by integration of the EQE curves.

b Calculated according to the equation HI = (PCE_Reverse_ − PCE_Forward_)/PCE_Reverse_^25^

External quantum efficiency (EQE) measurements (Fig. 2c) confirm the enhanced photoresponse of Cl24-F-based devices across the entire visible spectrum. The integrated *J*_SC_ values calculated from EQE spectra are consistent with the *J*–*V* measurements within 5% error, validating the accuracy of the performance parameters. Notably, Cl24-TCl delivers inferior device performance despite its larger dipole moment. This suggests that factors beyond molecular polarity dominate and require further investigation of energy level alignment, film morphology, charge transport, and interfacial charge dynamics.

Proper energy level alignment between the perovskite absorber, ETM, and metal cathode is essential for efficient charge extraction. The work functions and lowest unoccupied molecular orbital (LUMO) levels of the perovskite film and ETMs were determined using ultraviolet photoelectron spectroscopy (UPS, SI Fig. 2) and electrochemical measurements (SI Fig. 3), with the corresponding data summarized in SI Table 3 and 4. As illustrated in Fig. 2d, the perovskite film exhibits a work function of −4.09 eV and a LUMO level of −4.04 eV, whereas Cl24-TCl and Cl24-F show work functions of −4.85 eV and −4.36 eV, and LUMO levels of −4.29 eV and −4.20 eV, respectively. The LUMO offset between Cl24-F and the perovskite (0.16 eV) is smaller than that of Cl24-TCl (0.25 eV), enabling more efficient electron extraction while maintaining a sufficient driving force. In addition, the work function of Cl24-F (−4.36 eV) aligns more closely with Ag (−4.26 eV), promoting Ohmic contact formation, reducing interfacial energy losses,^26^ and minimizing non-radiative recombination, which enhances both *V*_OC_ and FF.

### 2.3 Film morphology and surface properties

**Fig. 3:**
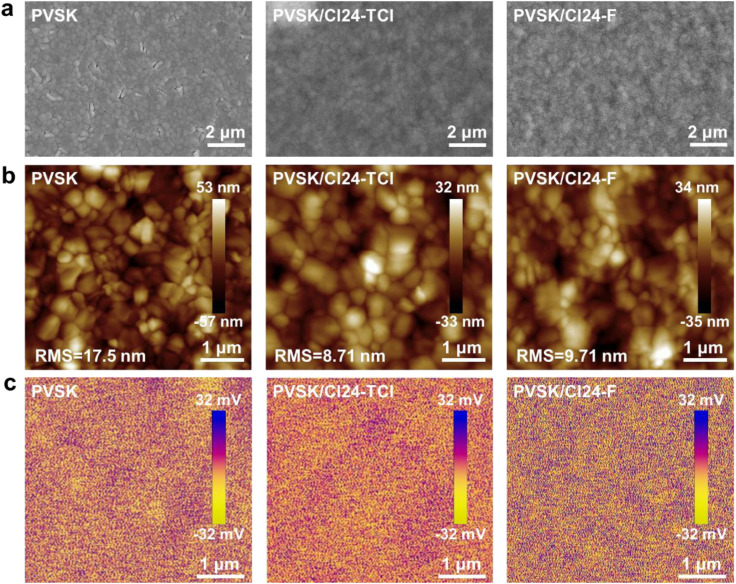
(a) Surface SEM images, (b) AFM height images, (c) KPFM surface potential maps of PVSK, PVSK/Cl24-TCl and PVSK/Cl24-F films.

The morphology of ETM films deposited on perovskite substrates critically influences charge extraction efficiency and interfacial recombination.^27^ Scanning electron microscopy (SEM) images (Fig. 3a) reveal distinct differences in film-forming behavior between the two molecules. Cl24-F forms a remarkably uniform and dense film with complete coverage of the underlying perovskite grains, whereas Cl24-TCl films display noticeable thickness variations and localized aggregation. Atomic force microscopy (AFM) analysis (Fig. 3b) quantifies these morphological differences. The pristine perovskite film exhibits a root-mean-square (RMS) roughness of 17.5 nm. After coating with Cl24-TCl, the RMS roughness decreases to 8.71 nm, indicating partial smoothing of the perovskite surface. In contrast, Cl24-F-coated films show an RMS roughness of 9.71 nm, which is comparable to Cl24-TCl, but with a more uniform surface texture and fewer pinholes. The smoother and more homogeneous surface provided by Cl24-F is expected to improve interfacial contact with the subsequently deposited metal electrode and reduce shunt pathways.^20^ Kelvin probe force microscopy (KPFM) was employed to investigate the surface potential distribution of the perovskite films with and without ETM coatings (Fig. 3c). Both Cl24-TCl and Cl24-F coatings result in uniform surface potential distributions, indicating homogeneous interfacial energetics across the film surface.^28,29^

The surface wettability of the ETM-modified perovskite films was evaluated through water contact angle measurements (SI Fig. 4). The pristine perovskite film exhibits a relatively hydrophilic surface with a contact angle of 34.8°, consistent with the presence of polar surface terminations.^30^ After coating with Cl24-TCl, the contact angle increases significantly to 87.7°, indicating effective shielding of the underlying perovskite by the hydrophobic organic layer. Cl24-F-coated films show an even higher contact angle of 95.2°, demonstrating superior hydrophobicity. This enhanced water repellency can be attributed to the intrinsically hydrophobic fluorinated terminal groups and the more uniform, pinhole-free film morphology. The higher contact angle of Cl24-F suggests it forms a more effective moisture barrier, advantageous for long-term device stability.^31^

Consistent with the higher water contact angle observed for the Cl24-F film, the thermal stability of the corresponding devices was subsequently assessed. Unencapsulated PSCs were subjected to thermal aging in ambient air (30 ± 5% RH, 85 °C), as presented in SI Fig. 5. After 912 h of aging, the Cl24-TCl-based device retained only 77% of its initial PCE, whereas the Cl24-F-based device maintained over 87% of its initial performance under identical conditions. This enhanced thermal resilience is primarily ascribed to the more hydrophobic character of the fluorinated terminal groups, which effectively retards moisture ingress and suppresses humidity-induced degradation of the perovskite layer. Furthermore, maximum power point (MPP) tracking measurements were carried out at 25 °C and 30% RH (SI Fig. 6). After 700 h of continuous operation, the Cl24-TCl-based device preserved only 83% of its initial efficiency, while the Cl24-F-based device retained over 90%. The superior operational stability observed for the Cl24-F-based cell is fully consistent with its greater hydrophobicity, as a more effective moisture barrier is established at the perovskite/ETM interface, mitigating interfacial degradation under prolonged illumination.

### 2.4 Charge transport properties

**Fig. 4:**
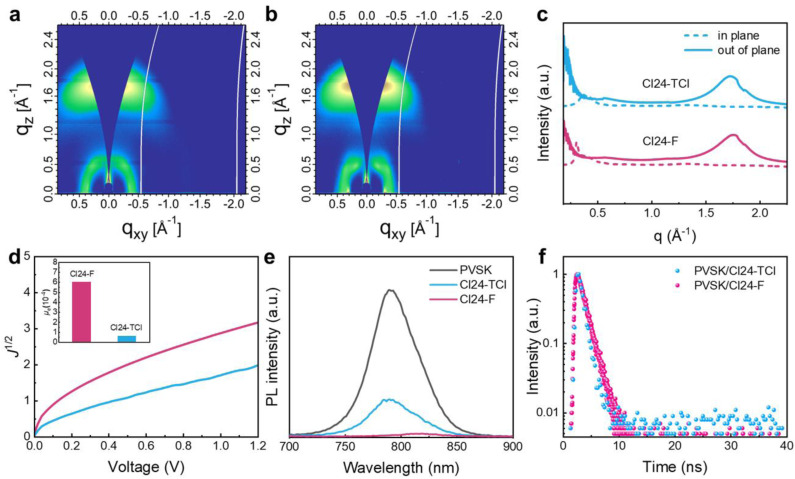
2D GIWAXS patterns of (a) Cl24-TCl, (b) Cl24-F films, and (c) the corresponding 1D profiles, (d) electron mobility curves for electron-only devices fabricated from neat Cl24-TCl and Cl24-F films, along with the corresponding values presented in the inset, (e) PL spectra of PVSK, PVSK/Cl24-TCl and PVSK/Cl24-F films, and (f) TRPL spectra of PVSK/Cl24-TCl and PVSK/Cl24-F films.

Grazing-incidence wide-angle X-ray scattering (GIWAXS) was performed to investigate the molecular packing and orientation of the ETM films. The 2D GIWAXS patterns and corresponding 1D line–cut profiles for Cl24-TCl and Cl24-F films are presented in Fig. 4a–c. Both molecules exhibit a pronounced (010) diffraction peak in the out-of-plane direction, corresponding to π–π stacking and characteristic of a face-on orientation preference. Such orientation is particularly beneficial for vertical charge transport, providing direct pathways for electron migration from the perovskite to the electrode. Quantitative analysis of the (010) peak (SI Table 5) reveals that Cl24-TCl has a larger crystalline coherence length of 18.57 Å compared with 16.87 Å for Cl24-F, yet it also exhibits a larger π–π stacking distance of 3.67 Å compared to 3.61 Å for Cl24-F. The shorter interchain distance in Cl24-F promotes stronger electronic coupling between adjacent molecular backbones,^32,33^ facilitating more efficient charge carrier hopping.

The molecular packing characteristics directly influence macroscopic charge transport. The electron transport characteristics of the non-fullerene ETM were evaluated through space-charge-limited current (SCLC) measurements using electron-only devices with the structure FTO/ETM/Ag. As shown in Fig. 4d, Cl24-F exhibits an electron mobility of 6.04 × 10^−4^ cm^2^ V^−1^ s^−1^, approximately one order of magnitude higher than that of Cl24-TCl (6.00 × 10^−5^ cm^2^ V^−1^ s^−1^). Conductivity measurements (SI Fig. 7) reveal a similar trend, with Cl24-F showing a conductivity of 4.00 × 10^−3^ S m^−1^, substantially higher than the 1.66 × 10^−3^ S m^−1^ measured for Cl24-TCl. The improved mobility and conductivity of Cl24-F, arising from its more compact π–π stacking and favorable face-on orientation, enable more efficient charge transport through the ETM, thereby reducing series resistance and enhancing the FF in PSC devices.^34^

The superior charge transport properties of Cl24-F also translate into more efficient interfacial charge extraction. Steady-state photoluminescence (PL) measurements were conducted to evaluate charge extraction at the perovskite/ETM interfaces. As shown in Fig. 4e, the pristine perovskite film exhibits a strong emission peak at 789 nm. Upon coating with Cl24-TCl, the PL intensity decreases substantially, indicating partial electron extraction. For the Cl24-F-coated film, the PL is almost completely quenched, demonstrating highly efficient charge transfer at the interface. Moreover, the emission peak of the Cl24-F-coated sample shows a distinct redshift to 819 nm, suggesting stronger electronic interaction between Cl24-F and the perovskite surface. Time-resolved photoluminescence (TRPL) spectroscopy was employed to probe the charge extraction dynamics (Fig. 4f and SI Table 6). For the perovskite/Cl24-TCl sample, the PL decay time was determined to be 2.84 ns. In contrast, the Cl24-F-coated film exhibited a much faster PL decay with a lifetime of only 1.34 ns. This acceleration of PL quenching demonstrates that Cl24-F enables more efficient electron extraction from the perovskite, consistent with its favorable molecular packing, higher electron mobility, and more intimate interfacial contact.

To directly probe the interaction between the ETMs and the perovskite surface, X-ray photoelectron spectroscopy (XPS) measurements were performed (SI Fig. 8 and Table 7). Relative to the pristine perovskite film, the Pb 4f peaks of the perovskite/Cl24-TCl and perovskite/Cl24-F films exhibit significant shifts toward lower binding energy, indicative of Lewis acid-base coordination between undercoordinated Pb^2+^ ions and the electron-donating moieties of both ETMs. This observation provides evidence for the passivation of Pb-related surface defects, consistent with the suppressed non-radiative recombination deduced from the TRPL results. To further elucidate the charge recombination and transport characteristics, dark *J*–*V* measurements were performed on the devices (SI Fig. 9). Under reverse bias, the Cl24-F-based device exhibited substantially suppressed leakage current compared to its Cl24-TCl counterpart, indicating that the denser and more uniform Cl24-F film effectively mitigates interfacial charge recombination and shunt pathways.^35,36^ In the forward bias region, the Cl24-F-based device displayed a steeper current rise, reflecting lower series resistance and more efficient charge transport. These observations are fully consistent with the higher electron mobility and conductivity of Cl24-F, corroborating its superior charge extraction capability.

The extraordinary difference in charge extraction kinetics between the two molecules reflects a synergistic combination of factors, including a more favorable energy level alignment that reduces the injection barrier, higher electron mobility that enables rapid transport of injected electrons away from the interface, more intimate interfacial contact due to superior film morphology, effective passivation of surface defects through interactions between the difluorinated terminal groups and undercoordinated Pb^2+^ sites, and reduced trap-mediated recombination at the interface. The enhanced electron extraction capability of Cl24-F minimizes carrier accumulation at the interface and suppresses non-radiative recombination losses, contributing to improved *V*_OC_ and FF in devices.

## 3 Conclusion

We systematically investigated two A–DA′D–A type NFAs, Cl24-TCl and Cl24-F, with distinct terminal electron-withdrawing groups as ETMs for inverted PSCs. Despite its higher dipole moment and more polarized charge distribution, Cl24-TCl yields inferior device performance compared to Cl24-F. Comprehensive characterization reveals that Cl24-F offers more favorable energy level alignment with the perovskite and Ag cathode, superior electron mobility and conductivity, and a pronounced face-on molecular orientation. TRPL measurements show accelerated electron extraction at the Cl24-F/perovskite interface with a decay time of 1.34 ns *versus* 2.84 ns for Cl24-TCl. These combined advantages enable Cl24-F-based devices to achieve a champion PCE of 24.18%, significantly outperforming Cl24-TCl-based devices (13.02%). This work establishes a clear structure–property relationship linking terminal group engineering to interfacial charge dynamics, demonstrating that balanced charge distribution, optimal film morphology, and strong interfacial interactions are key parameters for high-performance non-fullerene ETMs.

## Author contributions

F. P., X. L. and M. L. conceived the idea. F. P. and X. L. designed the project. Y. G. and X. L. synthesized Cl24-TCl and Cl24-F. F. P. and S. Z. fabricated the devices and conducted relevant characterizations. J. G. performed the DFT calculations. C. L. and J. X. offered helpful discussions. M. L. supervised the project. F. P., X. L. and C. X. wrote the manuscript with input from all authors.

## Conflicts of interest

There are no conflicts to declare.

## Supplementary Material

RA-016-D6RA02579J-s001

## Data Availability

The data supporting this article have been included as part of the supplementary information (SI). Supplementary information is available. See DOI: https://doi.org/10.1039/d6ra02579j.
