# Supplementary material for: Terminal group engineering of A–DA′D–A non-fullerene acceptors as electron transport materials for efficient inverted perovskite solar cells
Source: RSC Adv. 2026 May 19;16(29):26756–63. doi: 10.1039/d6ra02579j (PMC13187896; doi:10.1039/d6ra02579j)
Supplement: RA-016-D6RA02579J-s001 [file RA-016-D6RA02579J-s001.pdf]

Supplementary Information (SI) for RSC Advances.
This journal is © The Royal Society of Chemistry 2026

Supplementary Information

Terminal Group Engineering of A-DA'D-A Non-Fullerene Acceptors as Electron Transport Materials for Efficient Inverted Perovskite Solar Cells

Shengdong Zhao [a,†], Yufei Gong [b,†], Fei Pan[a,c,*], Junjiang Guo[c], Chengcheng Xie[a], Chenxing Lu[b], Xiaojun Li[b,*], Jingchao Xu[d,*], Menglan Lv[a,*]

[a] Engineering Research Center for Energy Conversion and Storage Technology of Guizhou, School of Chemistry and Chemical Engineering, Guizhou University, Guiyang, 550025, PR China

[b] Beijing National Laboratory for Molecular Sciences, CAS Key Laboratory of Organic Solids, Institute of Chemistry, Chinese Academy of Sciences, Beijing 100190, PR China.

[c] School of Chemical Engineering, Guizhou Institute of Technology, Guiyang 550025, PR China

[d] Dongfang Electric (Hangzhou) Innovation Institute Co., Ltd., Hangzhou, PR China

Corresponding authors: fpan@git.edu.cn (F. Pan), lixiaojun@iccas.ac.cn (X. Li), xc2009xust@163.com (J. Xu), mllv@gzu.edu.cn (M. Lv).

† The first two authors contributed equally to this work.

Experimental Section

Materials

Cesium iodide (CsI, 99.9%), dimethyl formamide (DMF, anhydrous) and dimethyl sulfoxide (DMSO, anhydrous) were purchased from Sigma-Aldrich. Formamidinium iodide (FAI, 99.5%), methylammonium iodide (MAI, 99.5%), lead iodide (PbI_2, 99.9%), lead chloride ($PbCl_2$, 99.9%), 1,3-Propanediammonium diiodide (PDADI) and C_{60} were purchased from Xi'an Yuri Solar Co. All materials were used as received without further purification. FTO substrates were purchased from Suzhou Shangyang Solar Technology Co.. Cl24-TCl and Cl24-F were synthesized according to the procedure reported in the literatures[1]. The detailed synthetic processes are described in the following **Supplementary Scheme 1**.

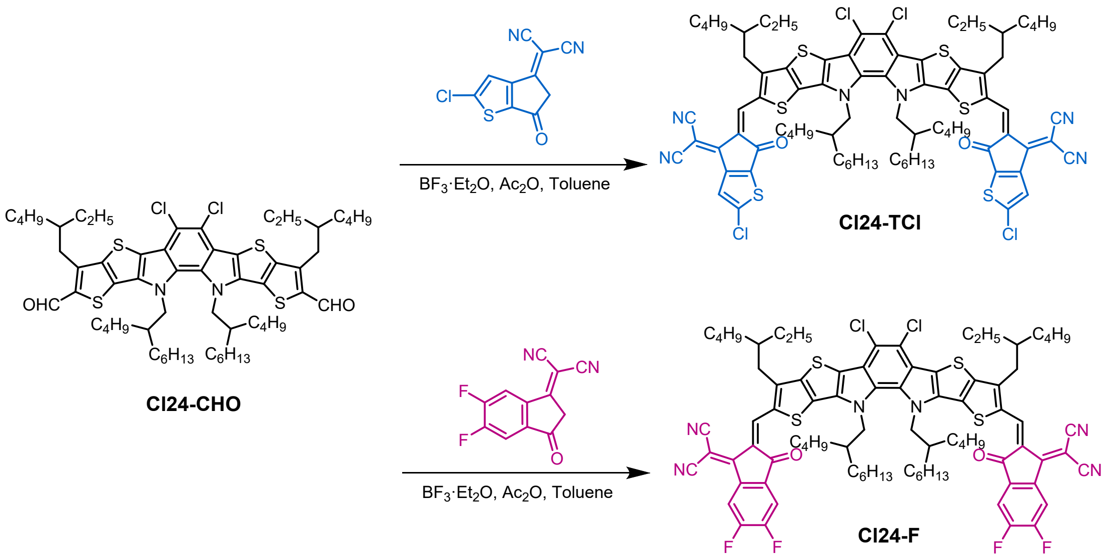

Supplementary Scheme 1. Synthetic procedures of Cl24-TCl and Cl24-F.

Density functional theory (DFT) calculations

For the molecular NFAs calculations, the geometry optimizations and vibrational frequency analyses were conducted using B3LYP functional with 6-31G(d,p) basis set as implemented in the Gaussian16 program. The electrostatic potential maps for NFAs were obtained at the same theoretical level.

Characterization of NFA and perovskite films

X-ray Photoelectron spectroscopy (XPS) data and Ultraviolet photoelectron spectroscopy (UPS) were measured by Kratos AXIS ULTRA DLD spectrometer. Non monochromatic He I (21.22 eV) source was used as an excitation source. Gold was used as reference. The bias voltage applied during the test is -9 V.

The HOMO and LUMO energy levels of NFA were determined via cyclic voltammetry (CV, GAMRY), with glassy carbon as the working electrode, platinum wire as the counter electrode and Ag/AgCl as the reference electrode. The scan rate is 0.1 V s^{-1} and the supporting electrolyte is the acetonitrile solution of tetrabutylammonium hexafluorophosphate with a concentration of 0.1 mol L^{-1}. The solutions of the NFAs were dropped onto the working electrode to form thin films, and ferrocene/ferrocenium (Fc/Fc^{+}) redox couple was used as the internal standard.

The atomic force microscopy, Kelvin probe force microscopy (KPFM) images were obtained by Bruker Dimension ICON. Steady-state photoluminescence (PL) spectra were carried out with Horiba Jobin Yvon system. The excitation wavelength was set to 490 nm, and the final spectra were obtained by averaging three scans.

The films tested in time-resolved photoluminescence (TRPL) were recorded on an Edinburgh Instruments FLS1000 fluorescence spectrometer equipped with a 450 W xenon lamp and a 475 nm picosecond laser as excitation sources.

Surface and cross-sectional morphology of the films and devices were examined by scanning electron microscopy (SEM, SU8010). For the cross-sectional image, the sample was coated with ca. 1 nm-thick gold using sputter to enhance the conductivity.

Device fabrication

The patterned FTO glass substrates were sonicated with detergent, deionized water, acetone and isopropanol for 30 min sequentially. After the ultraviolet ozone treatment for 30 min, a Ph-4PACz SAM (1 mg/mL) was then spin-coated at 3000 rpm. for 30 s and annealed at 100 °C for 10 min. $Cs_{0.05}FA_{0.9}MA_{0.05}PbI_3$ was prepared according to the stoichiometric ratio with solvent of DMSO: DMF (v/v = 1/5) as the perovskite precursor, with excessive 5% $PbCl_2$ and MAI addition. The 1.8 M

perovskite precursor solution was spin-coated at 5,000 rpm for 30 s with an acceleration of 5,000 rpm s^{-1} and at the 15 s, 800 μL diethyl ether poured onto the substrate. The perovskite films were annealed at 100 °C for 30 min in the N_2 atmosphere. 0.5 mg mL^{-1} PDADI was spin-coated at 5,000 rpm for 30 s and then annealed at 100 °C for 10 min. The NFAs were weighed under ambient conditions, dissolved in chloroform solvent within a N_2-filled glovebox, and stirred for 30 min, spin-coated onto the perovskite film at 3000 rpm. for 30 s. BCP (8 nm)/Ag (100 nm) were deposited by thermal evaporation under a pressure of 2×10^{-6} torr. The devices were controlled by a mask with an area of 0.1024 cm^2 during the current density-voltage (*J-V*) measurement.

Characterization of devices

The *J-V* curves of the perovskite solar cells were measured using a Keysight B2901A Source-Meter, with the scanning voltage from 1.22 V to -0.2 V in 20 mV step and delay time of 1 ms. The measurements were performed in a glove box filled with nitrogen (oxygen and water contents are smaller than 10 ppm). Enli-tech class AAA Solar Simulator (model, SS-X180R-3A) with a 450 W xenon lamp was used as the light source. The EQE responses were measured by Solar Cell Spectral Response Measurement System QE-R3-011 (Enli Technology Co., Ltd., Taiwan). The light intensity at each wavelength was calibrated with a standard single-crystal Si photovoltaic cell.

Contact angle (CA) test was conducted through OCA 25 with water and diiodomethane. Electron mobility (μ_e) was measured using the device architecture FTO/NFA/Ag. As shown in **Fig. 4d**, the mobilities were extracted using the Mott-Gurney law:

$$J = \frac{9\varepsilon_0\varepsilon_r\mu V^2}{8d^3}$$

Supplementary Figures

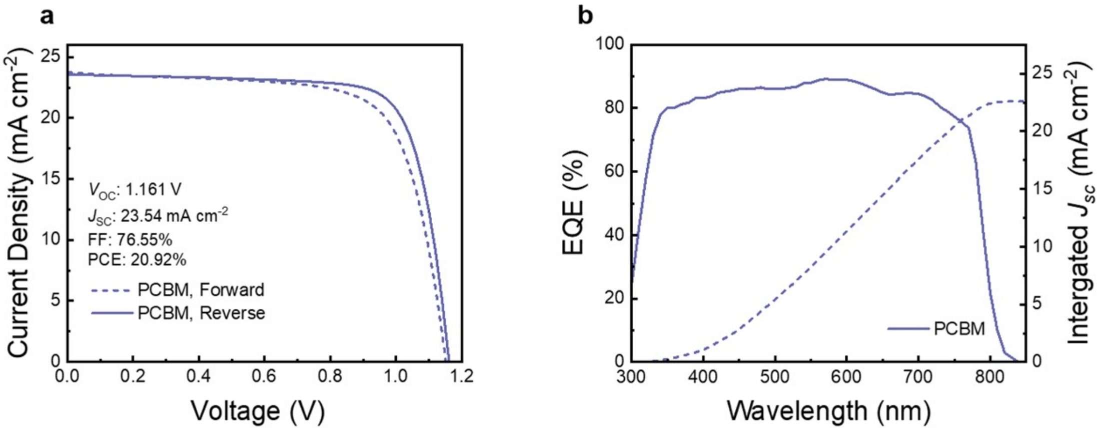

Supplementary Fig. 1. (a) Optimized *J-V* curves and (b) EQE spectra with the integrated J_{SC} values of the PSCs based on PCBM ETM.

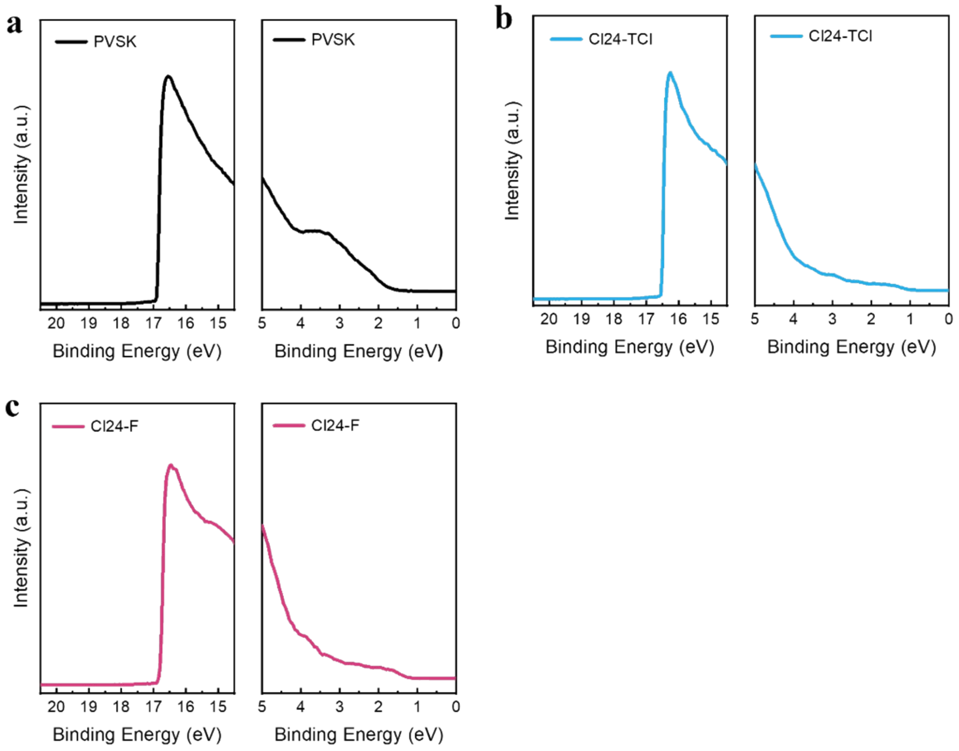

Supplementary Fig. 2. Ultraviolet photoelectron spectroscopy results of (a) PVSK, (b) Cl24-TCl, (c) Cl24-F films.

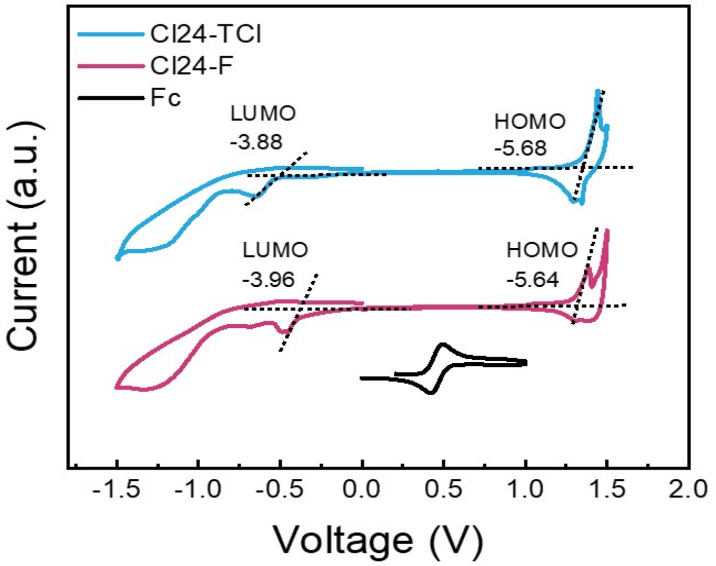

Supplementary Fig. 3. Electrochemical cyclic voltammetry scanning of Cl24-TCl and Cl24-F.

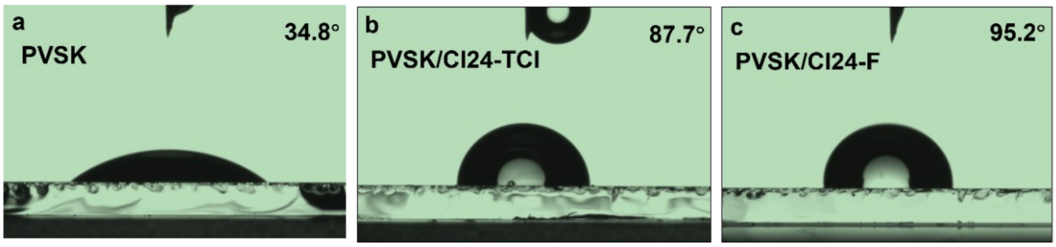

Supplementary Fig. 4. Contact angle measurement of (a) PVSK, (b) PVSK/Cl24-TCl, and (c) PVSK/Cl24-F films.

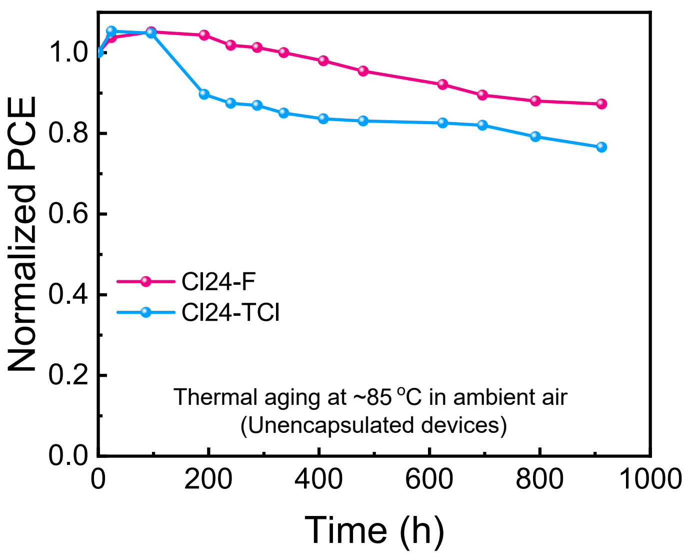

Supplementary Fig. 5. Thermal stability at 85 °C of the unencapsulated PSCs using Cl24-TCl and Cl24-F as ETMs.

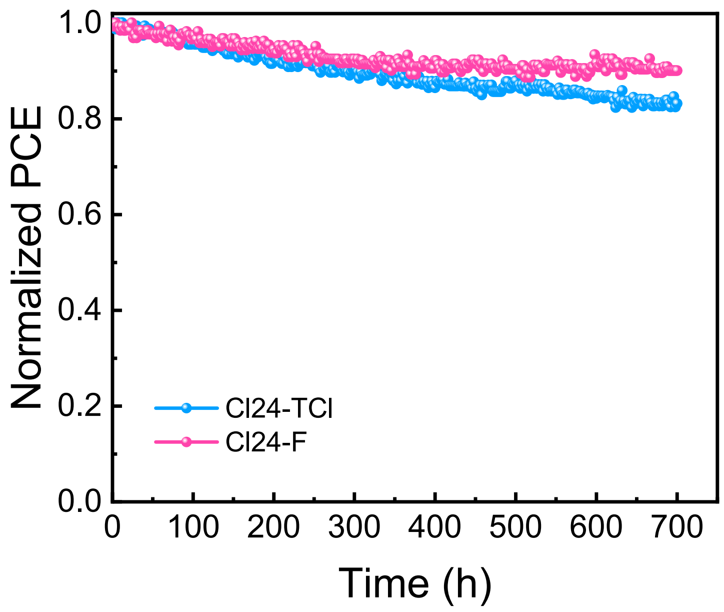

Supplementary Fig. 6. MPP tracking of the encapsulated PSCs using Cl24-TCl and Cl24-F as ETMs (LED lamp, 100 mW cm^{-2}, 25°C, 30% RH).

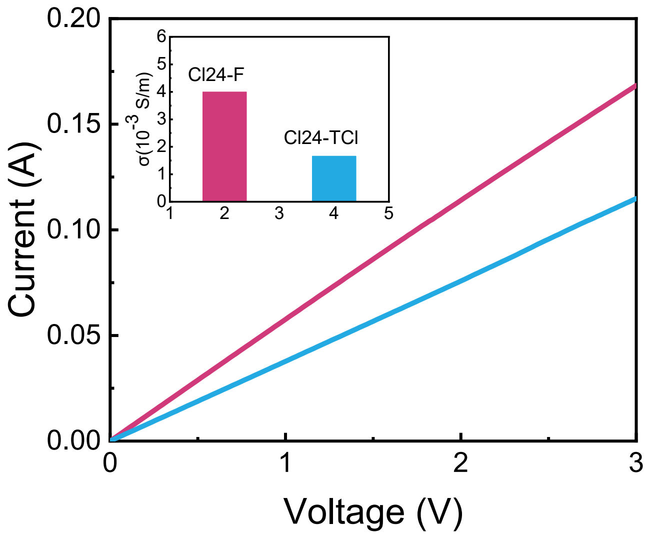

Supplementary Fig. 7. Conductivity curves for electron-only devices fabricated from neat Cl24-TCl and Cl24-F films, along with the corresponding values presented in the inset.

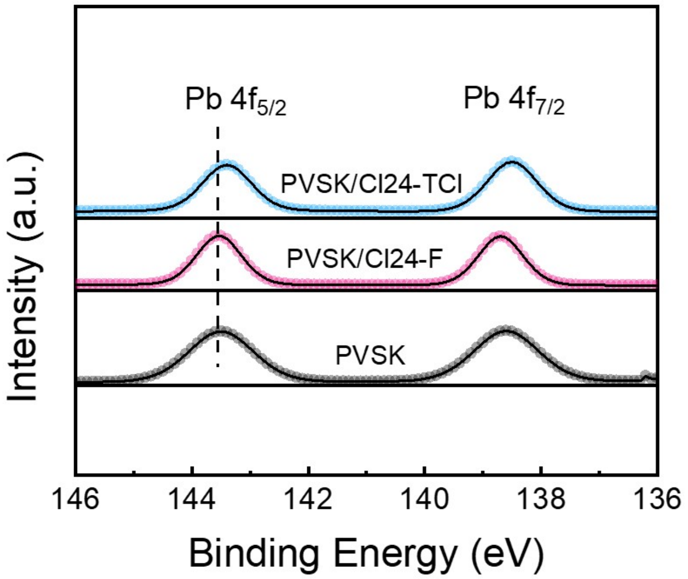

Supplementary Fig. 8. XPS spectra of Pb 4f for PVSK, PVSK/Cl24-TCl, PVSK/Cl24-F samples.

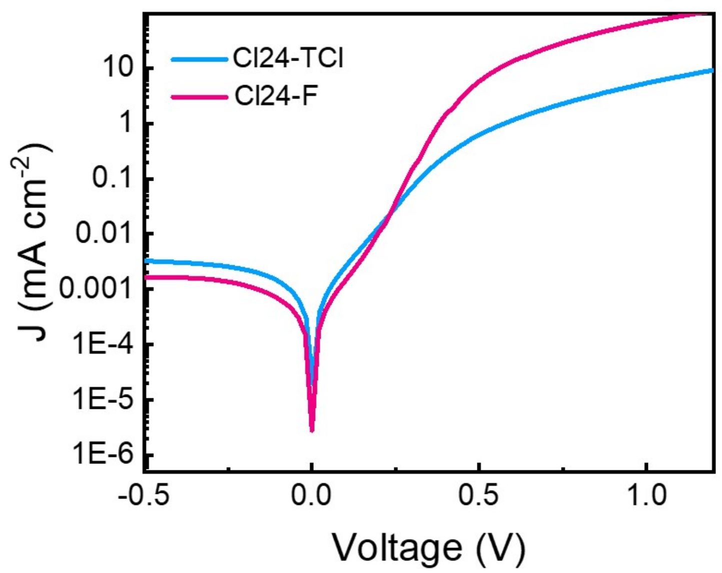

Supplementary Fig. 9. Dark *J-V* curve of PSCs with Cl24-TCl and Cl24-F as ETL.

Supplementary Tables

Supplementary Table 1. The electrostatic potential data of Cl24-TCl, Cl24-F.

NF ETM	Overall Average ESP [a] $\overline{V}$ (kJ mol^{-1})	Overall Variance [b] $\sigma 2$ tot (kJ^2 mol^{-2})	Balance of Charges [c] ν (arb. units)	Molecular Polarity Index [d] (MPI) (kJ mol^{-1})
Cl24-TCl	11.82	134.19	0.25	11.29
Cl24-F	4.55	131.43	0.24	10.46

a) $\overline{V}$ were calculated by $\overline{V} = \frac{1}{t}\sum_{k=1}^{t} V(r_k)$

b) $\sigma 2$ tot were calculated by $\sigma 2$ tot = $\sigma 2$ ++ $\sigma 2$ -= $\frac{1}{m}\sum_{i=1}^{m}\left[V(r_i) - \overline{V}_s^{+}\right]^2 + \frac{1}{n}\sum_{j=1}^{n}\left[V(r_j) - \overline{V}_s^{-}\right]^2$

c) ν were calculated by $\nu = \frac{\sigma_+^2 \sigma_-^2}{\left(\sigma_{tot}^2\right)^2}$

d) MPI were calculated by $MPI = \frac{1}{t}\sum_{k=1}^{t}\left|V(r_k)\right| \equiv \frac{1}{A}\iint_s |V(r)| dS$

Supplementary Table 2. Summary of average photovoltaic performance parameters of different ETMs-based perovskite solar cell devices based on 15 devices.

ETMs	V_{OC} (V)	J_{SC} (mA cm^{-2})	FF (%)	PCE (%)	J_{cal}[a] (mA cm^{-2})
PCBM	1.161 (1.158 ± 0.005)	23.54 (23.52 ± 0.38)	76.55 (74.84 ± 1.47)	20.92 (20.39 ± 0.47)	22.59
Cl24-TCl	1.136 (1.107 ± 0.025)	14.84 (14.24 ± 0.95)	77.26 (70.67 ± 4.84)	13.02 (11.16 ± 1.33)	14.25
Cl24-F	1.171 (1.167 ± 0.004)	24.56 (24.67 ± 0.28)	84.09 (82.04 ± 1.64)	24.18 (23.63 ± 0.44)	23.58

[a]The values were determined by integration of the EQE curves.

Supplementary Table 3. Optical and electrochemical properties of PVSK, Cl24-TCl and Cl24-F

Sample	E_{cutoff} (eV)	WF (eV)	E_{onset} (eV)	λ_{max} (nm)	E_g (eV)	E_{HOMO} (eV)	E_{LUMO} (eV)

PVSK	17.13	-4.09	1.52	789	1.57	-5.61	-4.04
Cl24-TCl	16.37	-4.85	1.01	789	1.57	-5.86	-4.29
Cl24-F	16.86	-4.36	1.35	819	1.51	-5.71	-4.20

$WF = h\nu - E_{\text{cutoff}}$, where $h\nu$ is the photon energy used in the UPS measurement

$E_{\text{HOMO}} = 21.22 \text{ eV} - (E_{\text{cutoff}} - E_{\text{onset}})$

Supplementary Table 4. Electrochemical properties of Cl24-TCl and Cl24-F determined by cyclic voltammetry.

ETM	Φ_{ox} [eV]	E_{HOMO} [eV]	φ_{red} [eV]	E_{LUMO} [eV]	E_g
Cl24-TCl	1.34	-5.68	-0.46	-3.88	1.80
Cl24-F	1.30	-5.64	-0.38	-3.96	1.68

Supplementary Table 5. Morphology parameters from GIWAXS fits to line profiles of NFA films.

NF ETM Film	FWHM ($Å^{-1}$)	d-spacing (Å)	CCL (Å)
Cl24-TCl	0.3045	3.67	18.57
Cl24-F	0.3351	3.61	16.87

Supplementary Table 6. Time-resolved photoluminescence (TRPL) fitting parameters for different ETLs in Fig. 4f. These parameters were extracted by fitting the TRPL decay curves to a biexponential function.

Sample	A_1	τ_1	A_2	τ_2	τ_{ave}
Cl24-TCl	229.12	0.16	1024.38	2.87	2.84
Cl24-F	250.68	1.34	77.12	1.34	1.34

Supplementary Table 7. Binding energy of lead species derived from peak fitting of the Pb 4f core-level XPS spectra in **Supplementary Fig. 7**.

Sample	Pb $4f_{5/2}$	Pb $4f_{7/2}$
PVSK	143.5	138.6
PVSK/Cl24-TCl	143.2	138.3

PVSK/Cl24-F	143.3	138.5

References

1. Y. Li, Y. Gong, X. Li, H. He, S. Qin, J. Zhang, J. Zhang, F. Pan, L. Meng and Y. Li, J. Mater. Chem. C, 2025, 13, 21205-21214.
